# A quantitative textual analysis of the subjective effects of ayahuasca in naïve users with and without depression

**DOI:** 10.1038/s41598-023-44193-5

**Published:** 2023-11-10

**Authors:** Lucas Cruz, Bheatrix Bienemann, Fernanda Palhano-Fontes, Luís Fernando Tófoli, Dráulio B. Araújo, Daniel C. Mograbi

**Affiliations:** 1 Department of Psychology, Pontifical Catholic University of Rio de Janeiro (PUC-Rio), Rio de Janeiro, Brazil; 2 Brain Institute, Federal University of Rio Grande do Norte (UFRN), Natal, Brazil; 3 Interdisciplinary Cooperation for Ayahuasca Research and Outreach (ICARO), Faculty of Medical Sciences, University of Campinas (UNICAMP), Campinas, Brazil; 4 Institute of Psychiatry, Psychology & Neuroscience, King’s College London, De Crespigny Park, PO Box 078, London, UK

**Keywords:** Neuroscience, Psychology

## Abstract

Ayahuasca is a brew with psychoactive properties that has been used as an entheogen for centuries, with more recent studies suggesting it is a promising treatment for some clinical disorders. Although there is an emerging scientific literature on its effects, to the best of our knowledge no study has explored the self-reported experiences of first-time ayahuasca users with quantitative textual analysis tools. Accordingly, the current study aimed to analyze the subjective experience of naive individuals with depression and healthy controls after consuming ayahuasca. For this purpose, responses from a subsample of participants from a previous clinical trial to open-ended questions regarding their experience with ayahuasca underwent textual analysis. Data from nine patients with treatment-resistant depression and 20 healthy individuals were included, and quantitative textual analysis was performed using IRaMuTeQ 0.7 alpha 2 and R 3.1.2. The analysis identified five clusters: alterations in the state of consciousness, cognitive changes, somatic alterations, auditory experiences, and visual perceptual content. Additionally, findings suggest specific features of the experience of people with depression with ayahuasca, such as increased aversive bodily reactions. The results are consistent with previous findings indicating central axes of the psychedelic experience, and may inform therapeutic approaches using ayahuasca.

## Introduction

Ayahuasca is a brew with psychoactive properties made from the decoction of Jagube vine (*Banisteriopsiscaapi*) and Chacrona leaf (*Psychotriaviridis*). Indigenous groups have consumed it for centuries as entheogens^1^, and in the past century syncretic religious organizations, such as *União do Vegetal*, *Barquinha*, and *Santo Daime*, have also used it ritualistically^2^. Clinical research on ayahuasca has indicated potential therapeutic effects for different mental health conditions and symptoms, such as panic-like and hopelessness symptoms^3^ and substance abuse disorder^4–6^. In addition, improvements in neuropsychological functioning have also been observed^7^.

Ayahuasca has shown promise for the treatment of some clinical disorders. In particular, depression is one of the conditions that may benefit the most from ayahuasca. Epidemiological data show that depression affects more than 300 million people worldwide^8^. Between 2010 and 2018, the incremental economic burden of adults with major depressive disorder (MDD) increased from $US236.6 billion to 326.2 billion. In this context, antidepressants, particularly selective serotonin reuptake inhibitors, have been largely prescribed, but their efficacy is discussed. For instance, a recent reanalysis of a network meta-analysis questions the efficacy of antidepressants for depression in adults, discussing whether they have stronger effects than placebo or not^9^. This highlights the urgent need for innovation in the management of depression, with more research needed into potential treatment alternatives.

In this context, recent studies with ayahuasca in depressive patients have shown potential therapeutic effects^10,11^. Palhano-Fontes^12^ assessed a sample of people with treatment-resistant depression who had previously attempted antidepressant therapy unsuccessfully previously. The study observed that depression severity was significantly reduced in participants who were dosed with ayahuasca, as assessed through scores on the Montgomery-Asberg Depression Rating Scale—MADRS^13^ and Hamilton Depression Rating Scale—HAM-D^14^. This reduction was observed one, two, and seven days after dosing when compared to placebo.

Unlike traditional pharmacological interventions, substances such as ayahuasca may be highly linked to characteristics of set (intrinsic subjective factors, such as expectation, preparation, personality, and intention) and setting (extrinsic factors, such as the surrounding environmental and social context of the experience^15^). Specifically for ayahuasca consumption, set and setting features may contribute positively to users’ acute experiences, with better mental health outcomes and increased well-being^16^. One way of exploring directly the effects of set is to have studies recruiting participants with and without clinical disorders, allowing the comparison of their subjective experiences.

Studies with ayahuasca, especially clinical investigations, have generally focused on quantitative measures of psychological characteristics. The integration with subjective aspects of the experience may be particularly relevant considering it has been argued these are necessary for the therapeutic effects of psychedelics^17^, but see^18^. Considering the studies that investigated subjective reports, Wolff et al.^19^ used qualitative content analysis to explore experiences after an ayahuasca ceremony, indicating that visual content may play an important role for therapeutic processes, with the possibility of users attributing symbolic meaning to them. This is in line with the work by Shanon^20^ exploring the epistemics of ayahuasca visions, suggesting they were intrinsically related to personal ideations, leading to various types of knowledge and teaching patterns. Beyond individual expectations, context has been shown to exert an important effect in subjective experiences. This has been explored in Israeli and Palestinians individuals, suggesting that sociopolitical group affiliation may affect the psychedelic experience in a relational sense^21^.

Findings from a thematic categorical analysis approach by Fernández et al.^22^ suggest that clinical and non-clinical groups may experience the subjective effects of ayahuasca in different ways. Clinical population reports had content such as biographical review, insights and experiences of emotions or death that may have high therapeutic value, while the content of healthy subjects was more related to thoughts about human culture, religion, and philosophy. Loizaga-Velder and Verres^23^ exploratory study suggests that ayahuasca may assist people with substance-abuse disorder to overcome psychological issues, for instance with higher integration of bodily and mental experience. Thus, exploring further how clinical and non-clinical groups respond to ayahuasca may bring important information for therapeutic approaches.

The analysis of qualitative data may also help revealing central axes of the psychedelic experience. Apud et al.^24^ investigated participants of a therapeutic center, mainly treated for substance use disorder (SUD), but also with other therapeutic demands. Their results suggest five components of the experience, which included emotional, corporal, spiritual/transcendental, personal elements and visions. Trichter et al.^25^ explored changes in spirituality in first time ayahuasca users, with their qualitative interviews indicating ten main themes in participants’ reports: presence of light/geometric patterns, sense of honor, respect, gratitude and/or awe, sense of connection, self-reflection and/or insights of personal life, spiritual experience, supernatural experiences, sense of peace and/or calm, healing, death/near-death experiences and desolation. Despite these studies, to the best of our knowledge, no previous research has used quantitative methods to analyze self-reported data of first-time ayahuasca users, including clinical and non-clinical participants.

In the last three decades, research on psychedelics has been conducted mostly in developed regions and high-income countries^26^. For substances such as dimethyltryptamine (DMT), mescaline, and psilocybin, this is paradoxical considering that their traditional use emerged in developing regions, particularly in Latin America. In the case of ayahuasca, in particular, its religious use is legally protected in Brazil, and the considerable number of users reinforces the need for more research into its effects. Further research conducted in developing regions is important not only to address these social needs, but also to investigate the extent to which the effects of psychedelics are universal.

Considering this, the current study explores the subjective experience of people with depressive and healthy controls who consumed ayahuasca. Specifically, a subsample of participants from the Palhano-Fontes et al.^12^ study answered open-ended questions about their experience with ayahuasca, with these data undergoing textual analysis. Although subjective experiences of users have been reported before, as indicated no one has investigated quantitatively the self-reported experiences of first-time ayahuasca users. Additionally, by exploring the perceptions of people with and without depression, the current work can shed light into the effects of set in the experience of ayahuasca, with particular implications for future clinical use of this substance.

## Methods

### Sample

To investigate the antidepressant effect of ayahuasca against a placebo, a randomized double-blinded placebo-controlled trial was conducted, with the results being reported previously elsewhere^12^. The primary investigation was a double-blind randomized placebo-controlled clinical trial aiming to test the antidepressant effects of ayahuasca on adults aged 18–60 with treatment-resistant depression. Participants were randomly assigned (1:1) to receive ayahuasca or placebo. All patients were naïve to ayahuasca, with no previous experience with any other psychedelic substance.

In the current study, data from nine patients with treatment-resistant depression and 20 healthy individuals were included. Only participants who took ayahuasca were included in the current analysis. All subjects in the study had no prior experience with ayahuasca. The exclusion criteria adopted were: diagnosis of current clinical disease (example: heart disease; diabetes) based on anamnesis, physical and laboratory examination; pregnancy; history of neurological diseases; history of bipolar affective disorder or schizophrenia; history of mania or hypomania; substance abuse. Patients were referred from psychiatric units of the Onofre Lopes University Hospital (HUOL), in Natal/RN, Brazil, and healthy volunteers were recruited through media and internet advertisements. All procedures took place at the HUOL. Socio-demographic characteristics of participants can be seen in Table 1.

**Table 1:** Socio-demographic data for participants (n = 29).

Variable	n
Gender (male/female)	11/18
Age*	32.2 (8.7)
Marital status (living alone/with others)	21/8
Ethnicity (White/Asian/Black or mixed)	16/2/11
Religion (Catholic/no religion/others)	11/10/7
Academic level (with/without higher education)	18/11
Income (high/low)	19/10
Cannabis user (yes/no)	5/24
Alcohol user (yes/no)	18/11
Tobacco user (yes/no)	4/25
Depression (yes/no)	9/20

*Mean (standard deviation).

### Procedures

Participants received a single dose of 1 mL/kg of ayahuasca. A single ayahuasca batch was used throughout the study, containing on average (mean ± SD): 0.36 ± 0.01 mg/mL of *N*,*N*-DMT, 1.86 ± 0.11 mg/mL of harmine, 0.24 ± 0.03 mg/mL of harmaline, and 1.20 ± 0.05 mg/mL of tetrahydroharmine, quantified by mass spectroscopy analysis. The batch was prepared and provided free of charge by a branch of the Barquinha church, Ji-Paraná, Brazil. The placebo was formulated as a liquid designed to mimic the color and flavor of ayahuasca.

Throughout the sessions, participants were given specific instructions to maintain silence, keep their eyes closed, and concentrate on their physical sensations, thoughts, and emotions. They were also provided with a predefined musical playlist to enhance their experience. In addition, two researchers were in an adjacent room to provide support whenever requested by the participants. The primary outcome measure was changes in the severity of depression, measured by the HAM-D scale, seven days after dosing (D7). The secondary outcome was changes in MADRS scores from baseline to 1 day (D1), 2 days (D2), and 7 days (D7) after dosing.

At the end of the dosing session, approximately 4 h after ayahuasca ingestion, when the acute effects had already ceased, we asked the participants to freely report their experience. Reports were recorded using an MP3-type recorder. These reports were later transcribed (Audiotext company, Curitiba/Brazil).

### Interviews, extraction of data and construction of textual corpus

Two researchers conducted the interviews, which were carried out right after the end of the acute effects of ayahuasca. Interviews had an average duration of 13.7 min and started with an open-ended question (“Can you please freely describe your experience?”). When participants gave brief answers, or when further clarification was needed, they were probed with follow-up questions (e.g. “I saw a person” “Was that person known to you? Can you please provide more details?”).

The transcribed reports were carefully reviewed and refined to ensure that no language or typing errors were present. To facilitate analysis by the software, dashes, quotation marks, and indentations were removed from the text. Reports had an average of 1002.6 words (for a model, see S1) and were categorized according to sociodemographic (sex, age, marital status, ethnicity, religion, educational achievement, and household income), and clinical variables (depressed/healthy individuals, past use of cannabis, alcohol or tobacco) of respondents. Sociodemographic and clinical variables were dichotomized to power the analyses. Missing values were classified as *null*.

### Data analysis

Exploratory and qualitative data analyses were conducted to generate familiarity with the content. Exploratory and qualitative data analyses were conducted to generate familiarity with the content. Interviews were read, one by one, by two co-authors (LC and BB). This was done to familiarize researchers with the context in which terms appeared, helping with the interpretation of the results provided by the automated analysis. IRaMuTeQ 0.7 alpha 2^27^ and R 3.1.2.^28^ were used to run the quantitative analysis. The analysis was implemented using text-segments (TS). Through TS, texts are divided by the context in which words appear and sized in accordance with the corpus extension. For this study, the default Iramuteq option was used (40 words per text segment, see S1). Reports were analyzed in their original language (Brazilian Portuguese), to avoid any loss of content and context, with the software output being translated into English for the presentation of results in the current article.

Descending Hierarchical Analysis (DHA, Reinert Method), Specificities and Correspondence Factor Analysis (CFA) were used. DHA organizes textual content in clusters with specific meanings, separated by the frequency of their vocabularies, similarity, and association. Specificities and CFA is a method that plays the role of associating texts with variables and indicate tendencies, proximities, and oppositions of the text segments (TS) in graphical visualization, locating those elements in a Cartesian graphic with factors based on their classifications and allowing graphic visualization of the co-occurrence between the words and the possible clusters they are part of Loubère^29^. The index of co-occurrence between the words (i.e., the relationship of the words between them and the communities formed by clusters composed of words that are most associated) are indicated by the specificities of the analysis. Specificities analysis highlights the co-occurrence between words and the communities formed by associated words. Words and categories were included in their respective classes by DHA based on the criteria of having a frequency greater than the mean of occurrences in the corpus and chi-square value with the cluster greater than 3.84 (the critical value for p < 0.05 with df = 1^30^). The words of interest (active forms) selected for analysis were adjectives, nouns, pronouns, verbs, adverbs, and forms not recognized by the IRaMuTeQ dictionary. In addition, when words had other associated forms (e.g. look, looking), the most frequent form was included in the graphic representation. Chi-square test was used to indicate the association between words and their clusters^29^. Cramer’s V was reported as a measure of effect size for the association^31^. α was set at 0.001 to avoid inflation of type I error.

### Ethical issues

The authors assert that all procedures contributing to this work comply with the ethical standards of the relevant national and institutional committees on human experimentation and with the Helsinki Declaration of 1975, as revised in 2008. The study was approved by the Research Ethics Committee of the Onofre Lopes University Hospital (# 579.479), and all subjects provided written informed consent prior to participation. This study was registered on 26/09/2016 at http://clinicaltrials.gov (NCT02914769).

## Results

### Descending hierarchical analysis

The analysis by DHA retained 75.03% of the total corpus, a percentage indicated as acceptable for the corpus to be considered for this type of analysis. The corpus was divided into 869 TS, relating 2968 words that occurred 29,029 times (mean of occurrence for TS = 33.41). Of these, the active forms formed 1829 words, with 691 words with a frequency equal to or greater than 3.

**Figure 1:**
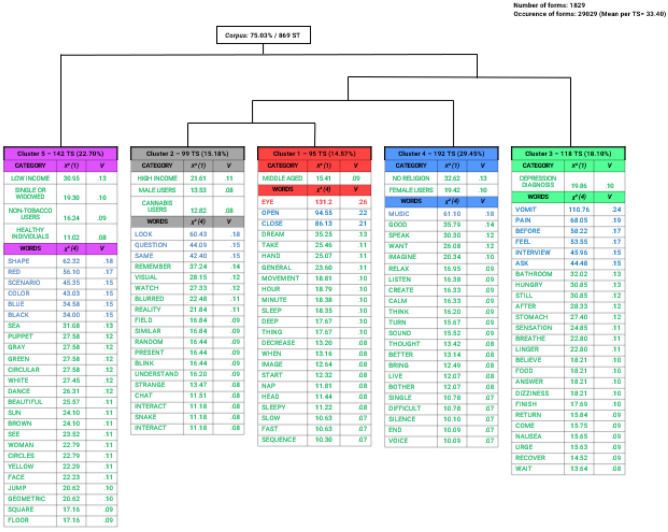
Dendrogram with words significantly associated (p < 0.001) with each cluster (max. words = 25) in interviews (n = 29) with individuals who consumed ayahuasca, including both volunteers (n = 20) and those with depression (n = 9), with an average of 1002.6 words per report. Large (red), medium (blue) and small (green) effect sizes, according to Kim^31^.

As seen in dendrogram form (Fig. 1), DHA resulted in five clusters of words. Initially, the clusters were grouped into distinct branches: with the first one consisting only of Cluster 5 (‘Visual perceptual content’—206 TS and 22.13% of total forms classified); the second was divided into two other branches with Cluster 3 (‘Somatic alterations’—118 TS and 18.10%) isolated, and all the other clusters together. Of these remaining clusters, Cluster 4 (‘Auditory experiences’—192 TS and 29.45%) splits first, with the last split separating Clusters 2 (‘Cognitive changes’—95 TS and 14.57%) and 1 (‘Alterations in the state of consciousness’—99 TS and 15.18%). For the association between words and clusters (degrees of freedom = 4), considering the five words with the highest association in each cluster, Cramer’s V indicated small, medium, and, particularly in Cluster 2, large effect sizes.

Figure 1 also shows the association of participants’ characteristics with each cluster. Cluster 1 was associated with middle-aged participants [χ^2^ (1) = 15.41; p < 0.001; V = 0.09], while Cluster 2 was associated with high-income [χ^2^ (1) = 21.61; p < 0.001; V = 0.11], male gender [χ^2^ (1) = 13.53; p < 0.001; V = 0.08] and cannabis users [χ^2^ (1) = 12.82; p < 0.001; V = 0.08]. Cluster 3 was associated with depression diagnosis [χ^2^ (1) = 19.86; p < 0.001; V = 0.10]. Cluster 4 was associated with female participants [χ^2^ (1) = 19.42; p < 0.001; V = 0.10] and those without religion [χ^2^ (1) = 32.62; p < 0.001; V = 0.13]. Cluster 5 was associated with low income [χ^2^ (1) = 30.95; p < 0.001; V = 0.13], single or widowed marital status [χ^2^ (1) = 19.30; p < 0.001; V = 0.10], non-tobacco users [χ^2^ (1) = 16.24; p < 0.001; V = 0.09] and healthy (non-depressed) individuals [χ^2^ (1) = 11.02; p < 0.001; V = 0.08].

### Correspondence factor analysis

**Figure 2:**
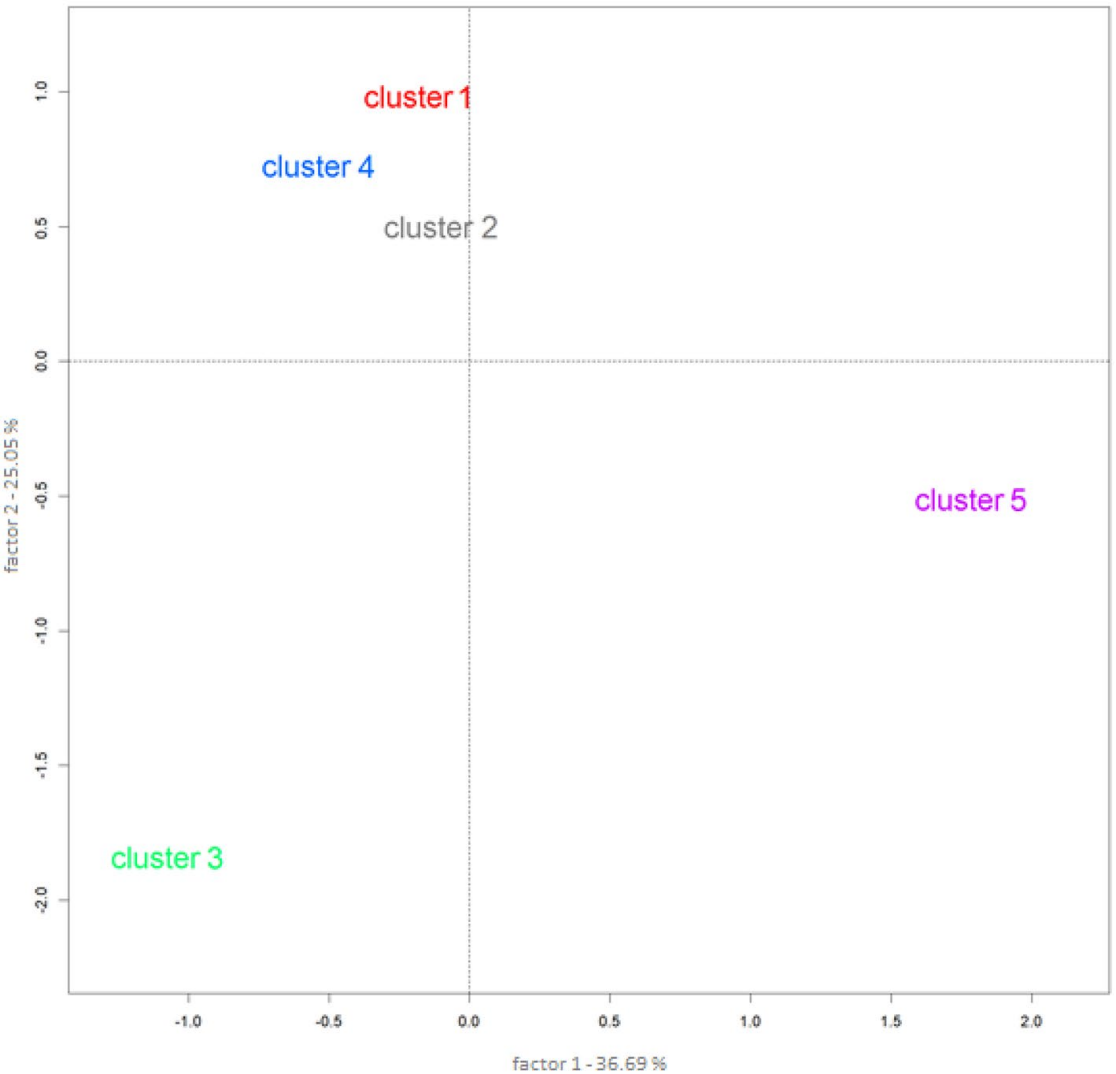
Relationship between clusters.

Results of correspondence factor analysis (CFA) can be seen in Fig. 2 (see also S2). The CFA indicated that the clusters are divided mainly into three areas, with Clusters 1, 2, and 4 being strongly related to each other, while Clusters 3 and 5 are isolated.

### Specificities analysis

**Figure 3:**
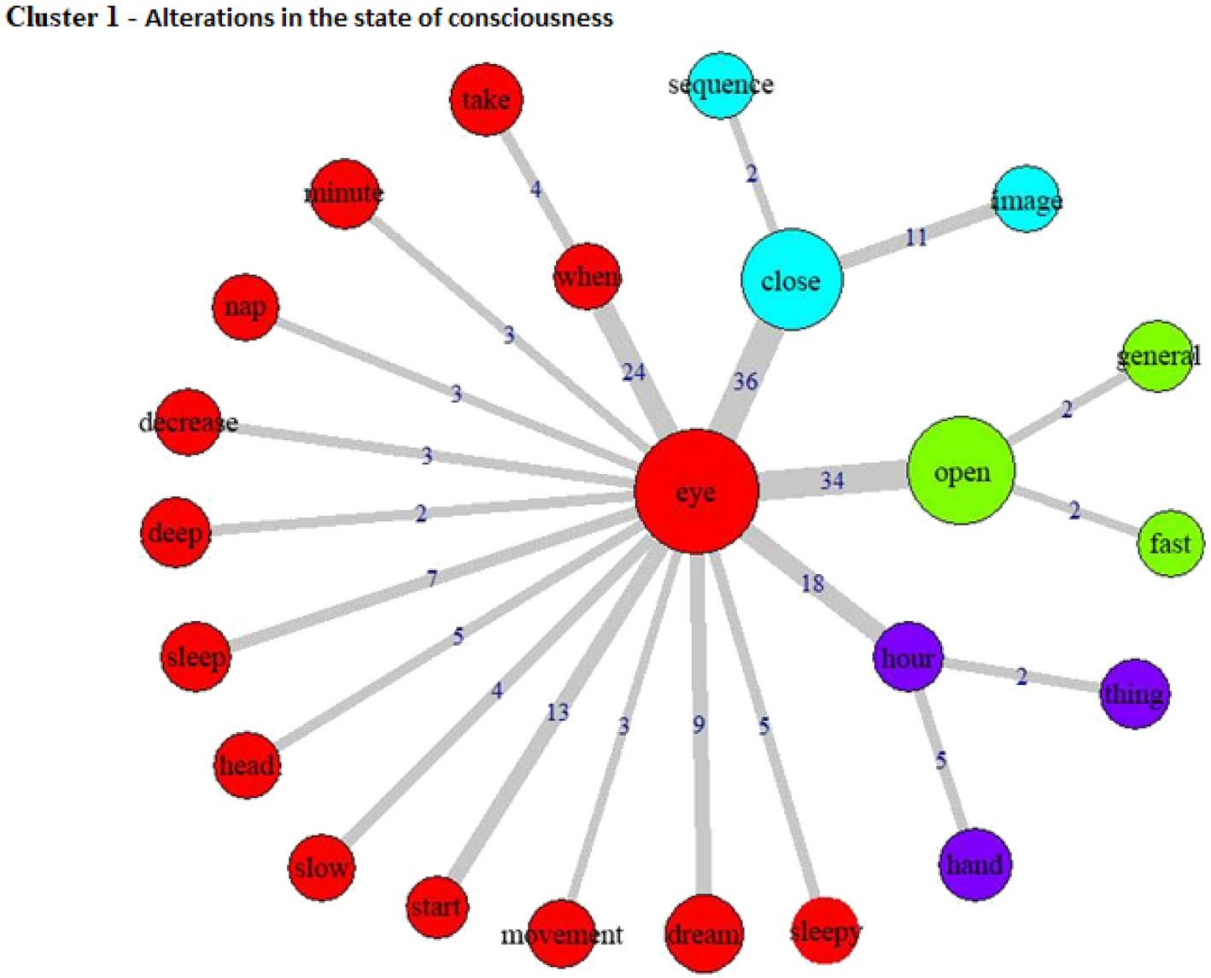
Co-occurrence and communities for words in Cluster 1.

**Figure 4:**
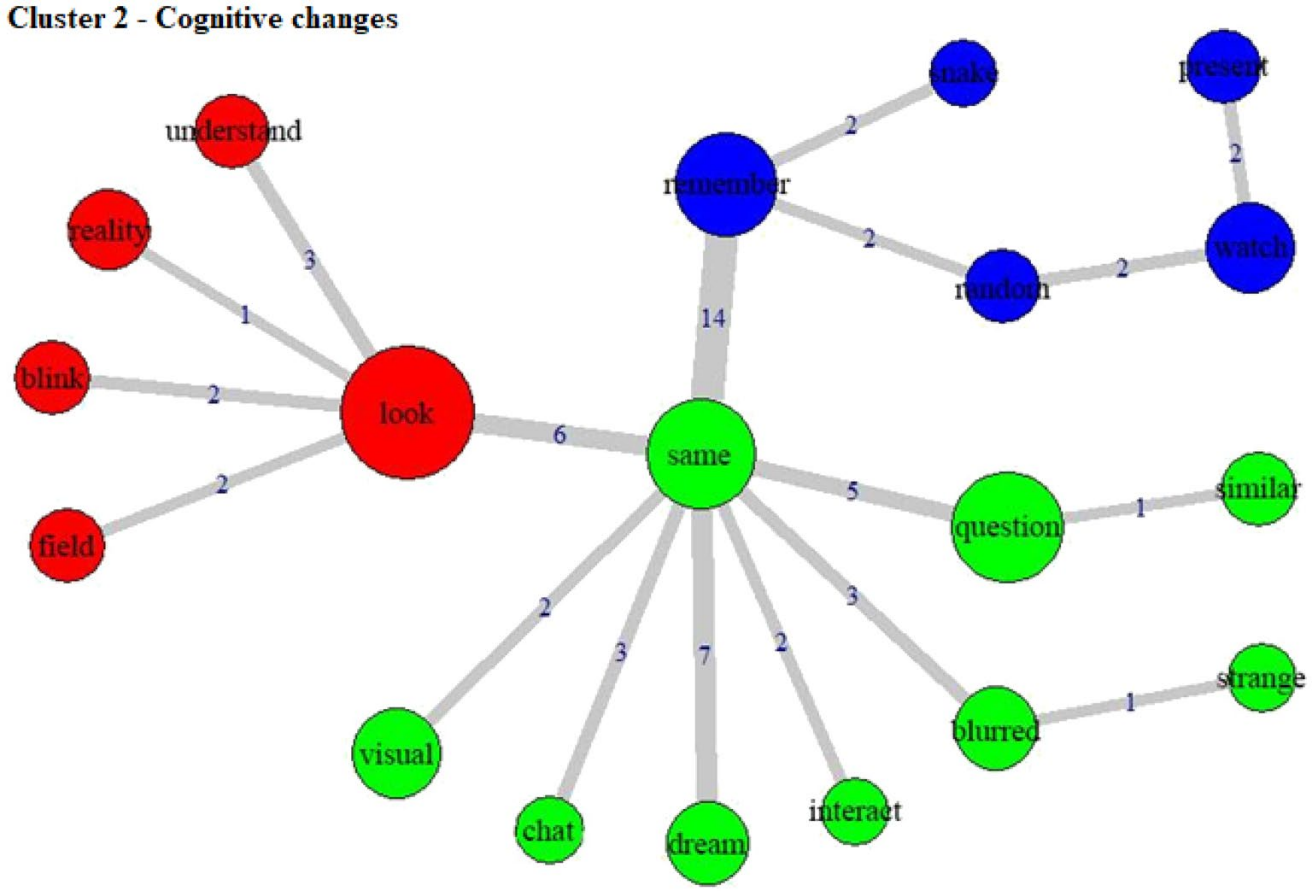
Co-occurrence and communities for words in Cluster 2.

**Figure 5:**
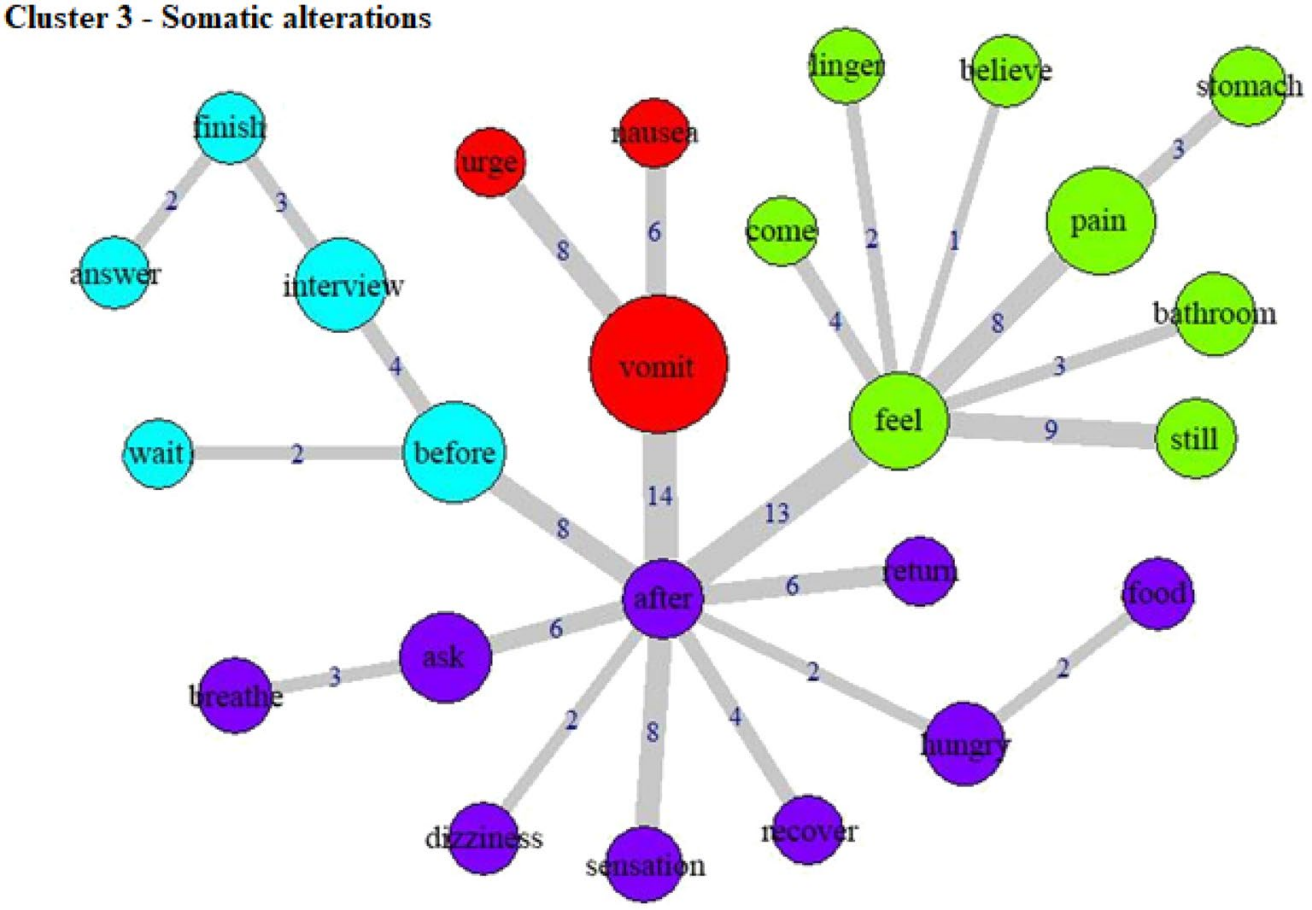
Co-occurrence and communities for words in Cluster 3.

**Figure 6:**
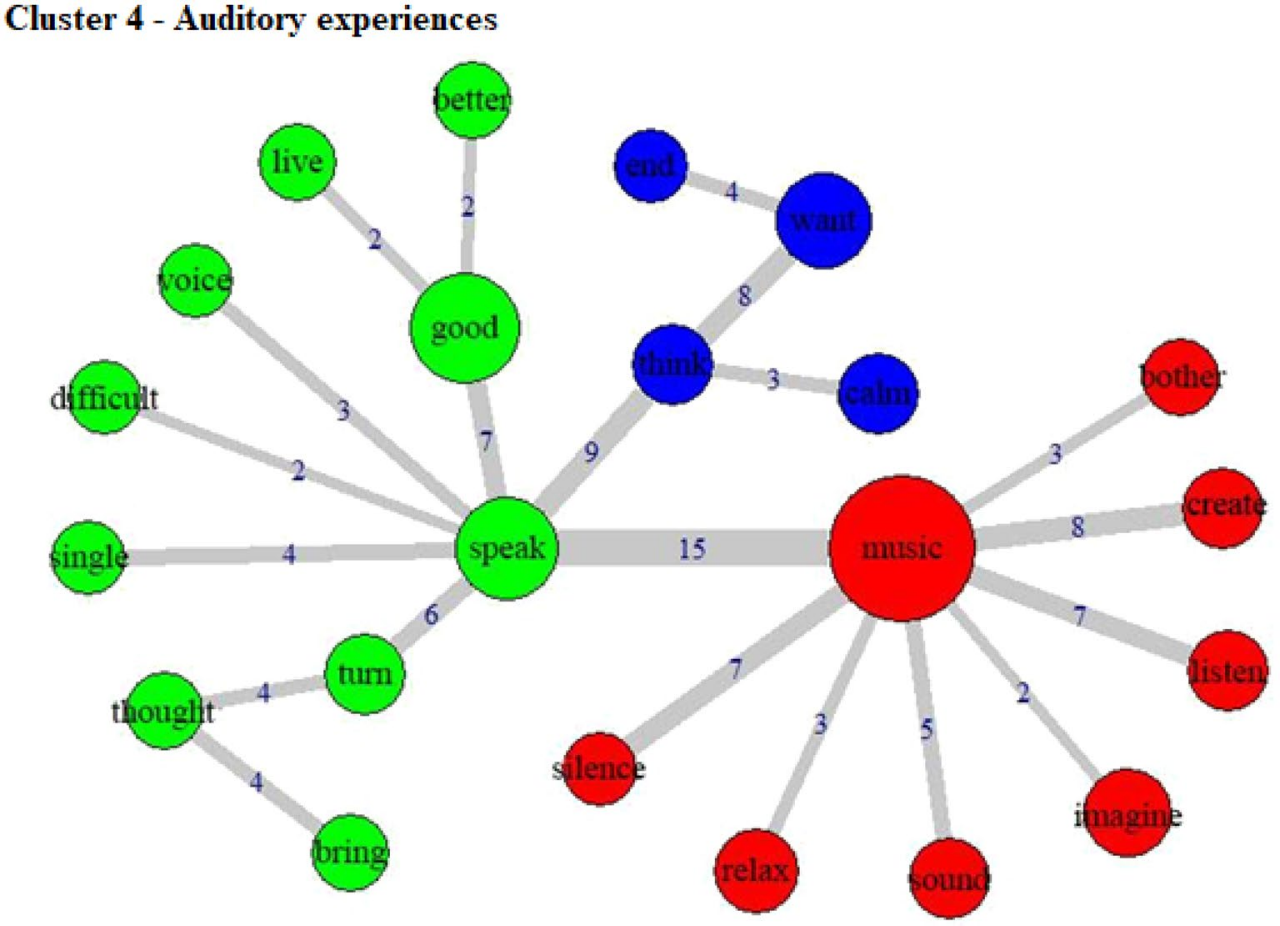
Co-occurrence and communities for words in Cluster 4.

**Figure 7:**
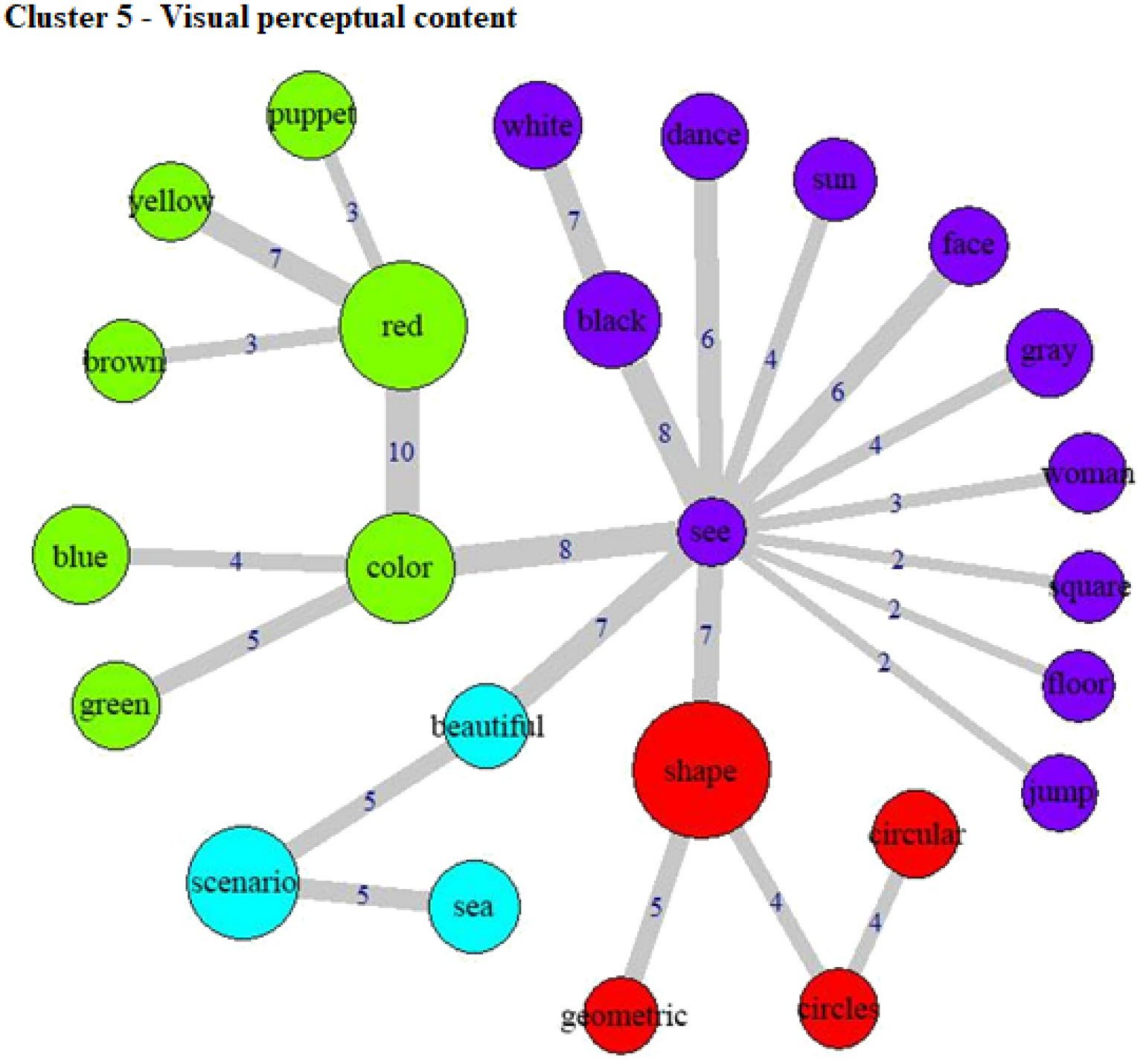
Co-occurrence and communities for words in Cluster 5.

The specificities analysis, indicating the index of co-occurrence between the words, can be seen in Fig. 3 (Cluster 1), Fig. 4 (Cluster 2), Fig. 5 (Cluster 3), Fig. 6 (Cluster 4), and Fig. 7 (Cluster 5).

## Discussion

In this study, we sought to analyze subjective aspects of the experience of people with depression and healthy controls after the consumption of a single dose of ayahuasca. The textual analysis, which congregate words repeated with high frequency during the participants’ reports describing the experience, identified five clusters. Broadly speaking these clusters refer to experiencing an altered state of consciousness, encompassing cognitive changes, somatic alterations, auditory experiences, and visual perceptual content. Each of these clusters showed specific associations with sociodemographic (e.g. age, gender, income, marital status) or clinical variables (depression diagnosis, substance use), but effect sizes were generally small.

Cluster 1 (14.5% of the TS) seems to indicate the onset and experience of an altered state of consciousness. The importance of the visual component at the beginning of the experience is notable^32^, which seems to be the central mediator of consciousness during this adaptive process. A central feature of the ayahuasca experience is that, according to user reports, visual hallucinations can be “switched on” or “off” by closing or opening the eyes. This highlights the crucial role of visual processing in the altered states of consciousness induced by ayahuasca consumption, which is also reflected in the strong association between the word “eye” and “open” and “close” in Cluster 1. It has been suggested that ayahuasca visions are often coupled with insights and topics with high personal relevance, including philosophical, metaphysical and spiritual epiphanies^20^, potentially contributing to the therapeutic effects of the substance^19^. These alterations in consciousness states can also be perceived initially as a decrease in the level of consciousness, which may explain the presence of words like “sleep”, “slow” and “nap”, in addition to content suggesting dream-like experiences (“dream”) and changes in time perception (“hour”). Middle-aged participants showed a significant association with this cluster, with a small effect size, which may suggest more marked consciousness alterations in naïve older participants, but without further data that remains speculative.

Cluster 2 (15.1% of the TS) includes a number of cognitive processes, through verbs such as “remember”, “question”, “look” and “understand”. It may indicate a sensory-perceptive integration of experience, with the association between “look” and “reality” or “look” and “understand”. The use of psychedelic substances may lead to an intense modification in one's perception of reality^33^, which is reflected in this cluster through words such as “blurred” or “strange”. Furthermore, a central word in this cluster word co-occurrence graph was “same”, which can mark an attempt to compare the perceived reality with an ordinary state of consciousness. This cluster is closely linked to Cluster 1, with shared characteristics of altered states of consciousness and their perception. Small effect sizes were observed for associations between this cluster with male, high income, and cannabis-user participants, which may reflect possible cognitive biases in these groups (e.g. more rumination in cannabis users^34^, but further research in this area is required to establish more robust conclusions.

Auditory characteristics of the experience and setting are highlighted in Cluster 4 (which gathered 29.4% of the TS), with words such as “music”, “speak”, “listen”, “sound”, “silence”, and “voice”. Most words in this cluster are associated with music, emphasizing the essential role of the music setting in shaping the experience^35^. In the word co-occurrence graph, it is possible to see how participants reacted positively or negatively to music (through words like “relax” or “bother”, for example). Speech was also an essential component of the experience. Some word associations can help us to understand speech as a process of understanding and dealing with the experience during it, such as links between “speak”—“good”—“better” or “speak”—“think”—“calm”. These auditory elements were particularly present in the reports of female participants and those without religion, which suggests these setting features were more salient for these groups. These findings also underscore the potential significance of spoken guidance provided during psychedelic experiences.

Cluster 3 (18.1% of the TS) includes words such as “vomit”, “pain”, “bathroom”, “hungry”, “stomach”, “breathe”, and “dizziness”, describing somatic aspects of the experience, particularly aversive feelings. That is in line with the purgative characteristic of ayahuasca, with users often going through difficult phases of the experience at its initial stages. Notably, the only category associated with this cluster was a diagnosis of depression. This may indicate a higher occurrence of negative somatic feelings in people with depression, consistent with the idea that depression is not only characterized by ideational, but also somatic symptoms (be, 2006). By contrast, this may represent cognitive biases in people with depression^36^, leading to more negative evaluation, recall, and reporting of experiences.

Cluster 5 (22.7% of the TS) seems to describe specific visual content. The word “see” is quite central in the co-occurrence graph, being linked to “color”, which then connects to specific colors. Human-like figures (“puppet”, “woman”) and their potential actions (“dance”, “jump”) are also represented. It is interesting to highlight the presence of neutral and positive biases (for example, the word co-occurrence graph reveals a link between “see” and “beautiful”) in this cluster, given that it is associated with people without depression. This may indicate, in contrast with Cluster 3, more benign experiences in healthy participants. This cluster is also associated with low income, living alone, and not using tobacco. Further studies may explore whether this represents a sampling artefact or if these associations are consistent.

The current findings are in agreement with those reported by Apud et al.^24^, who also recruited a clinical group (SUD). The five clusters indicated in their work show some overlap with the results reported here, for instance the ‘visual perception content’ and ‘somatic alterations’ clusters are linked to their ‘visions’ theme and ‘corporal experiences’ themes. Similar findings have also been reported by first-time ayahuasca users without clinical conditions^25^. It is possible that these core features of psychedelic experience can be measured by specific questionnaires. For instance, psychometric analysis indicates that the 5D-ASC questionnaire^37^ has a factor linked to Vigilance Reduction (VIR), which may capture phenomena described in Cluster 1, containing words such as “sleep”, “slow”, “nap”, and “dream”. Similarly, Cluster 4 has words such as “music”, “speak”, “listen”, “sound”, “silence”, and “voice” that are potentially related to a factor measuring Auditive Alteration (AVE). Future studies should explore the relationship between psychometric scales and verbal reports of experiences, trying to identify to which extent common dimensions of the psychedelic experience are assessed by existing instruments, leading to their refinement and development of new measurements.

In contrast to previous research^24^, current findings include a distinct cluster centered on auditory experiences, which encompasses both music and speech-related information, but lack a significant number of reports describing emotional and spiritual/transcendental aspects of the ayahuasca experience. This may be linked to characteristics of setting, with the current study being conducted in a university hospital, as opposed to previous investigations that took place in ritualistic^19^ or psychotherapy settings^24^.

In addition to potential setting effects, findings from the current study also support the idea that the set, specifically previous emotional state of participants, is linked to a more or less challenging experience. For example, Cluster 3 (‘somatic alterations’), describing mainly negative physical experiences, has a stronger association with individuals of depression, while Cluster 5 (‘visual perceptual content’), with more neutral and positive content, is more prevalent in reports of individuals without depression. This is in line with previous studies indicating that belonging to a clinical group modulates the experience with ayahuasca, for example with the study by Fernandez et al.^22^ suggesting that reports by people with depression focused on topics with higher therapeutic value in comparison to healthy individuals.

As indicated, the need of subjective effects of psychedelics for therapeutic efficacy has been debated. Recent research on non-hallucinogenic psychedelic analogs with therapeutic potential has suggested that neural plasticity and neurotrophic factors may be fostered independently of subjective experience^38,39^. While our study explored subjective states through quantitative analysis self-reports, it did not seek a direct comparison between subjective experience and therapeutic potential. Current results do indicate that the subjective state (depression, in this case) appears to influence how individuals assess and remember their experience. Whether attributed meaning has causal potential or is an epiphenomenon, it is undeniable that human beings live in the context of their symbolic representations, so it is unlikely that future therapeutic perspectives will reach its full potential by ignoring this fundamental dimension of human experience.

The current study has some important limitations. The sample investigated was limited in size, which may have reduced statistical power and ability to generalize results. Nevertheless, the number of participants recruited is consistent with that reported in other experimental studies in which psychedelic substances are administered to participants (e.g. Ref.^40^ [*n* = 27]; Ref.^41^ [*n* = 29]; Ref.^11^ [*n* = 17]). Additionally, in the scope of the current work, it was not possible to explore the relationship between specific symptoms and profiles of depression with the subjective experience of consuming ayahuasca. Future studies should investigate this further, given the potential clinical benefits of ayahuasca for people with depression^12,42^.

In summary, this study is the first to employ quantitative methods to analyze the subjective experiences of people with depression and healthy controls after the consumption of ayahuasca in a clinical setting. Findings highlight certain features of the psychedelic experience, such as visual content, effects on cognition, and somatic alterations, as well as specific aspects of how people with depression react to ayahuasca, such as increased aversive bodily reactions. Future studies should try to combine subjective reports with physiological data, aiming to elucidate specific mechanisms of the cognitive changes, including therapeutic effects, caused by ayahuasca. Given the context sensitivity of psychedelic experiences, studies in different settings may also help clarifying the extent to which contextual factors affect the perception of users of their experience.

## Data Availability

The dataset used for the current study is available from the corresponding author on reasonable request.
